# A 48-class handwritten dataset for the endangered chakma language

**DOI:** 10.1016/j.dib.2026.112796

**Published:** 2026-04-22

**Authors:** Jannatul Ferdeous, Md. Abdul Kayum, Ahmed Islam, Md Roman Nihal, ASM Shihavuddin, Muhammad Abul Hasan

**Affiliations:** a Department of Computer Science and Engineering, Green University of Bangladesh, Purbachal American City, Kanchon 1460, Dhaka, Bangladesh; b Department of Electrical and Electronic Engineering, Green University of Bangladesh, Purbachal American City, Kanchon 1460, Dhaka, Bangladesh; c Department of AI and Data Science, Green University of Bangladesh, Purbachal American City, Kanchon 1460, Dhaka, Bangladesh

**Keywords:** Handwritten character recognition, Chakma script, Low-resource language, Indigenous language, Digitization, Bangladesh

## Abstract

This article describes a comprehensive handwritten character dataset for the endangered Chakma language, primarily spoken in the Chittagong Hill Tracts of Bangladesh. The dataset comprises 37,708 processed RGB images, standardized to 40 × 40 pixels, covering 48 distinct classes that include 38 letters and 10 numerals. Data collection involved manual input from a diverse demographic of native speakers in the Rangamati and Khagrachhari districts, utilizing standardized form to capture authentic stylistic variability. The raw handwritten samples were subsequently digitized using high-resolution scanning, followed by automated cropping and resizing scripts to generate a uniform, machine-learning-ready format. This open-access resource addresses the scarcity of digital tools for indigenous scripts and can be utilized for training Handwritten Character Recognition (HCR) models, developing synthetic fonts via generative networks, and conducting linguistic analysis of Chakma handwriting characteristics.

Specifications Table

**Table:** 

Subject	Computer Sciences
Specific subject area	Handwritten Character Recognition, Machine Learning Data, Linguistic Analysis, Endangered Language Informatics
Type of data	Image Processed (Cropped, Resized)
Data collection	Manual collection using standardized forms. Contributors included students, graduates, and working adults. Forms were digitized using a high-resolution scanner. Custom Python scripts were used for automated cropping and resizing of individual characters based on grid coordinates.
Data source location	Rangamati and Khagrachhari, Chittagong Hill Tracts, Bangladesh.
Data accessibility	Repository name: Mendeley Data Data identification number: 10.17632/rzjphbvz9f.6 Direct URL to data: https://data.mendeley.com/datasets/rzjphbvz9f/6 [1] Instructions: The dataset is open-access. Users can download the data directly via the URL.
Related research article	None

## 1 Value of the Data

• The dataset provides a comprehensive resource for the underrepresented Chakma language, supporting efforts to mitigate language loss through digitization.
• These data can be used to evaluate and compare Deep Learning architectures (such as CNNs and ViTs) specifically for low-resource scripts.
• The samples, collected from diverse groups (students, workers) across multiple regions, ensure a high degree of stylistic variability, providing a robust resource for training models to generalize across diverse penmanship styles.
• This resource enables the development of interactive educational applications to teach Chakma literacy by facilitating real-time feedback on handwriting.
• The wide variety of captured styles can be utilized by Generative Adversarial Networks (GANs) to synthesize new, natural-looking Chakma computer fonts.
• The standardized format (40 × 40 pixels, RGB) allows for immediate integration into computational pipelines without requiring extensive preprocessing.
• To establish robust baseline benchmarks, we recommend evaluating models using a standard stratified 80–20 train-test split or a 5-fold cross-validation approach to ensure proportional representation across all 48 classes.

## 2 Background

**Fig. 1:**
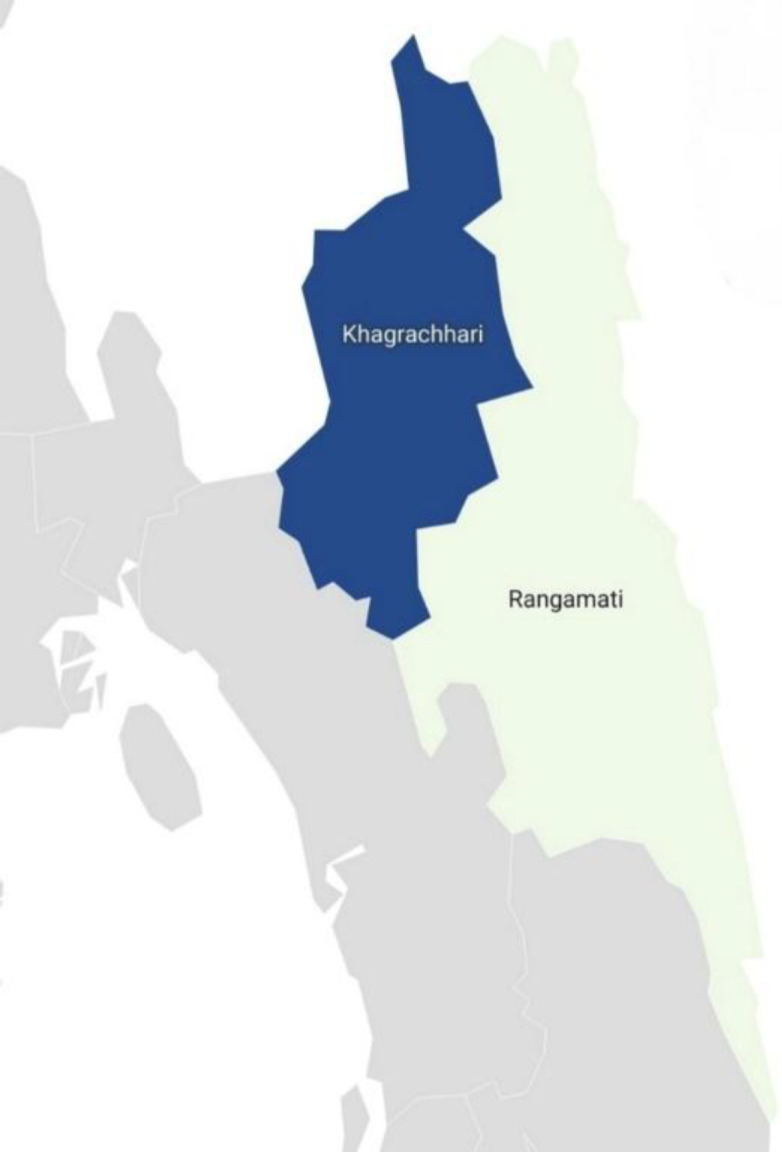
Map of Bangladesh highlighting the Rangamati and Khagrachhari districts, the primary habitat of the Chakma people.

The Chakma language (ISO 639–3 code: CCP) is one of the most widely spoken indigenous languages in Bangladesh [2,3]. Geographically, the Chakma people primarily inhabit the Chittagong Hill Tracts in the southeastern Bangladesh districts of Rangamati and Khagrachhari, as highlighted in Fig. 1. Despite its significance, the language faces severe endangerment due to sociolinguistic shifts toward Bangla [4,5]. Consequently, there is an urgent need for digital preservation tools to safeguard the script [6].

Current digital resources for Chakma are scarce. Although recent work by [7] introduced a dataset of 47,000 samples, it was collected exclusively via digital on-screen input. This methodology introduces a gap in ecological validity, as the kinematics of writing on a smooth glass surface differ significantly from traditional pen-on-paper handwriting regarding friction and stroke connectivity. The present dataset was curated to address this limitation by compiling 37,708 samples strictly from physical writing forms, capturing the natural noise and variability inherent in real-world documents.

## 3 Data Description

The Chakma Handwritten Character Dataset comprises a total of 37,708 RGB images, covering all 48 unique character classes (38 letters and 10 numerals) of the Chakma language.

### 3.1 Data representation

The dataset consists of individual image files. Computationally, each image is represented as a 3D matrix of dimensions 40 × 40 × 3. The pixel intensity values range from 0 to 255. The complete dataset represents a tensor of shape (N, H, W, C), specifically (37,708, 40, 40, 3), where N is the number of samples, H and W are the spatial dimensions, and C is the channel count.

### 3.2 Directory structure

The dataset is organized using a standard hierarchical folder structure:

**Root Directory:** ChakmaLipī Dataset

**Class Folders:** 48 sub-directories, each corresponding to a specific class label.

**Image Files:** Each class folder contains approximately 780 to 800 unique .png images.

A detailed summary of the distribution is provided in Table 1, and the complete mapping of directory class labels to their corresponding Chakma characters is presented in Table 2.

**Table 1:** Summary of the dataset distribution by category.

Category	Classes	Images per Class	Total Images
Letters	38	781	29,678
Numerals	10	803	8030
**Total**	**48**	**-**	**37,708**

**Table 2:** Mapping of directory class labels to Chakma characters.

Directory Name	Category	Description
digit_1	Numeral	Chakma Digit Zero ()
digit_2	Numeral	Chakma Digit One ()
digit_3	Numeral	Chakma Digit Two ()
digit_4	Numeral	Chakma Digit Three ()
digit_5	Numeral	Chakma Digit Four ()
digit_6	Numeral	Chakma Digit Five ()
digit_7	Numeral	Chakma Digit Six ()
digit_8	Numeral	Chakma Digit Seven ()
digit_9	Numeral	Chakma Digit Eight ()
digit_10	Numeral	Chakma Digit Nine ()
Character_1	Letter	Chakma Letter Kaa ()
Character_2	Letter	Chakma Letter Khaa ()
Character_3	Letter	Chakma Letter Gaa ()
Character_4	Letter	Chakma Letter Ghaa ()
Character_5	Letter	Chakma Letter Ngaa ()
Character_6	Letter	Chakma Letter Caa ()
Character_7	Letter	Chakma Letter Chaa ()
Character_8	Letter	Chakma Letter Jaa ()
Character_9	Letter	Chakma Letter Jhaa ()
Character_10	Letter	Chakma Letter Nyaa ()
Character_11	Letter	Chakma Letter Ttaa ()
Character_12	Letter	Chakma Letter Tthaa ()
Character_13	Letter	Chakma Letter Ddaa ()
Character_14	Letter	Chakma Letter Ddhaa ()
Character_15	Letter	Chakma Letter Nnaa ()
Character_16	Letter	Chakma Letter Taa ()
Character_17	Letter	Chakma Letter Thaa ()
Character_18	Letter	Chakma Letter Daa ()
Character_19	Letter	Chakma Letter Dhaa ()
Character_20	Letter	Chakma Letter Naa ()
Character_21	Letter	Chakma Letter Paa ()
Character_22	Letter	Chakma Letter Phaa ()
Character_23	Letter	Chakma Letter Baa ()
Character_24	Letter	Chakma Letter Bhaa ()
Character_25	Letter	Chakma Letter Maa ()
Character_26	Letter	Chakma Letter Yaa ()
Character_27	Letter	Chakma Letter Raa ()
Character_28	Letter	Chakma Letter Laa ()
Character_29	Letter	Chakma Letter Waa ()
Character_30	Letter	Chakma Letter Saa ()
Character_31	Letter	Chakma Letter Haa ()
Character_32	Letter	Chakma Letter Yyaa ()
Character_33	Letter	Chakma Letter Aa ()
Character_34	Letter	Chakma Letter E ()
Character_35	Letter	Chakma Letter I (Alternative of )
Character_36	Letter	Chakma Letter I ()
Character_37	Letter	Chakma Letter U ()
Character_38	Letter	Chakma Letter Lhaa ()

## 4 Experimental Design, Materials and Methods

**Fig. 2:**
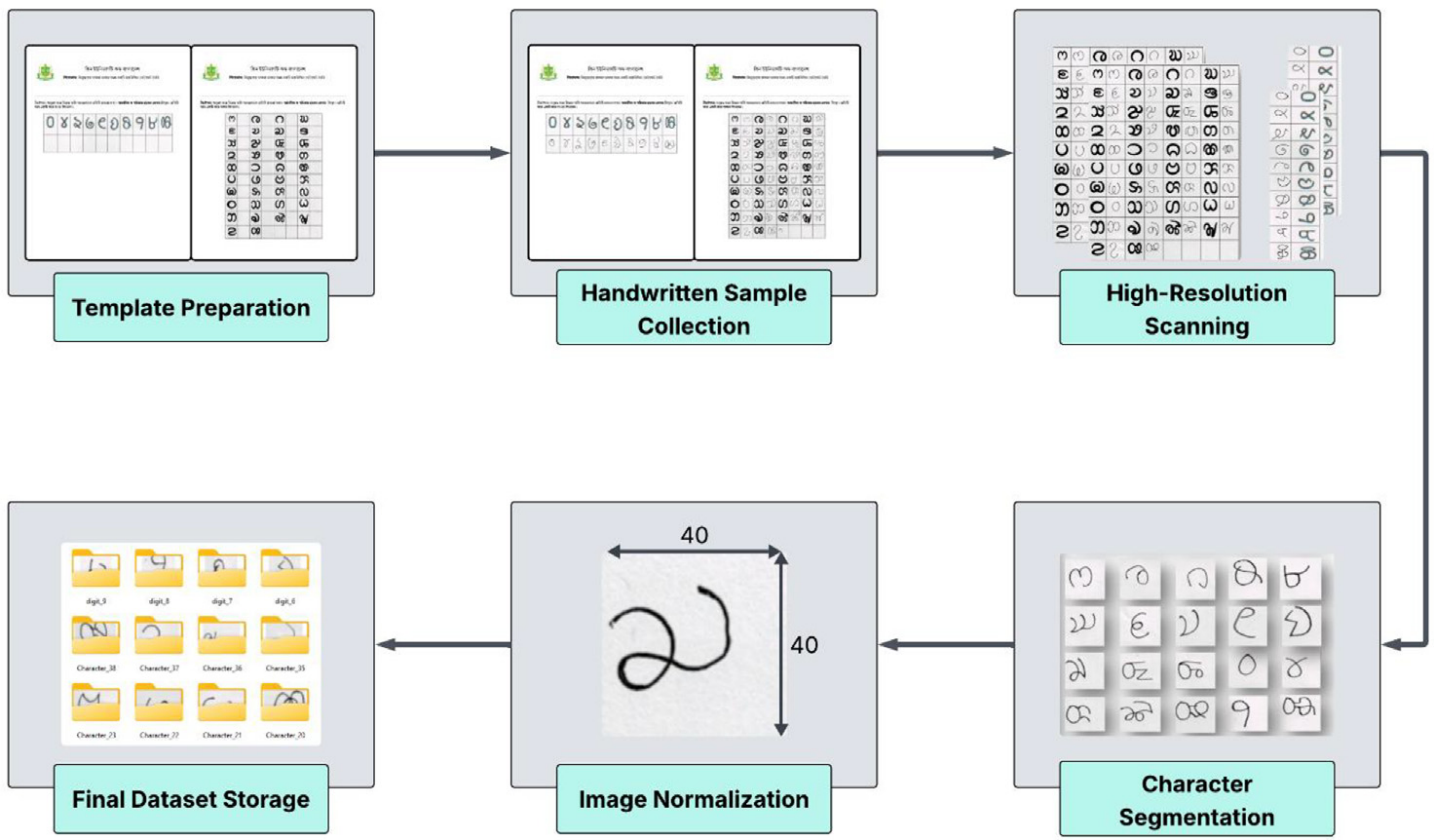
Workflow of the data collection and dataset creation.

The overall workflow adopted for data collection and dataset creation is summarized in Fig. 2.

A total of 1120 native Chakma speakers contributed to the dataset. The participants included 61% males and 39% females, ranging in age from 18 to 50 years. The demographic was drawn from across the local Chakma community, comprising 78% students and 22% working professionals. This ensured the capture of variability influenced by personal traits such as age, literacy, and motor skills. Each participant submitted one complete 48-class form.

The foundational letters and numeral set were sourced from the official Chakma textbook, Shanggu. This set comprises 38 fundamental letters and 10 numerals (0 to 9), resulting in a total of 48 distinct classes.

**Fig. 3:**
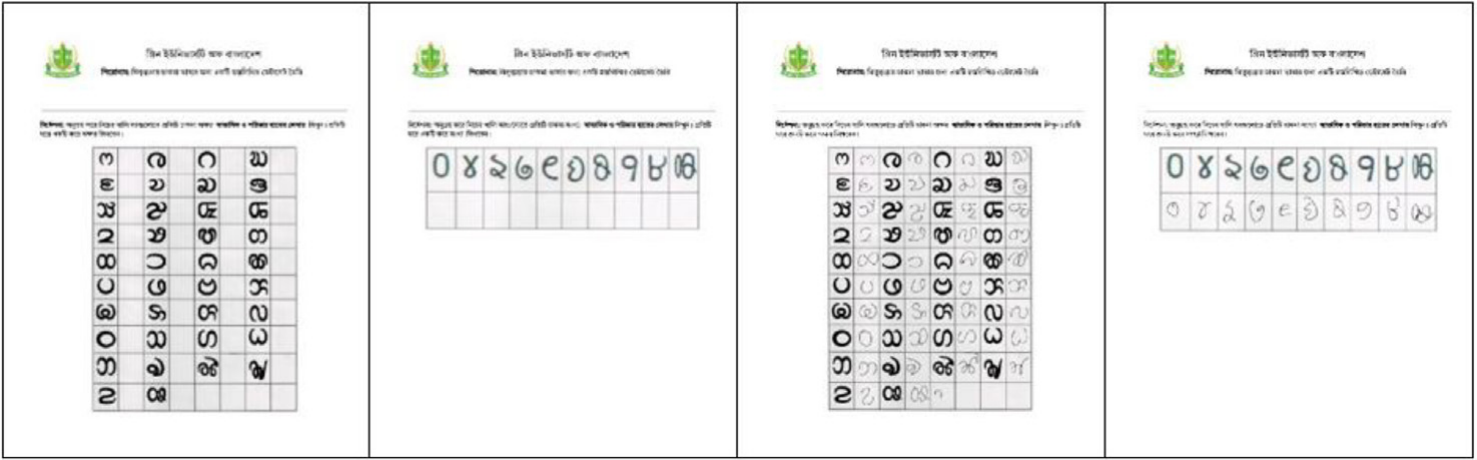
Data collection forms used to obtain Chakma handwritten characters.

**Fig. 4:**
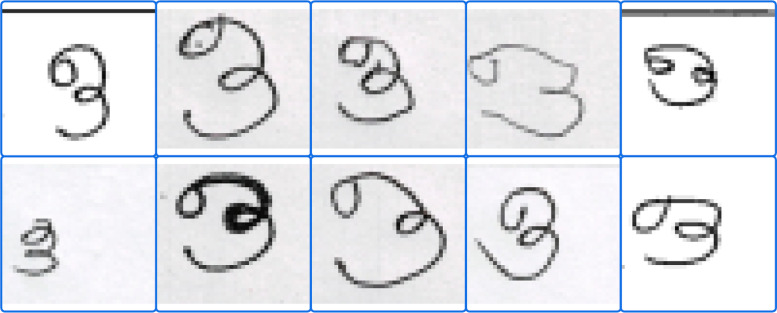
Sample images demonstrating the diversity of handwriting styles for a single character.

Fig. 3 shows the structured data collection form that was employed to rigorously capture the complete set of 48 classes from each participant. Furthermore, Fig. 4 highlights the dataset's richness by illustrating the intra-class variability.

To maintain high data quality, all completed forms were digitized using a Brother DCP-T310 scanner at a resolution of 300 DPI. This process yielded a collection of scanned sheets that served as the raw input.

### 4.1 Dataset creation

The raw scanned forms underwent a rigorous process of verification, automated cropping, and resizing to transform the collected sheets into a structured image dataset.

First, each scanned form was manually verified to ensure legibility and completeness of all handwritten entries. Forms containing incomplete or unclear samples were excluded from further processing.

Character-level image extraction was performed automatically using a Python script. The script employed a predefined set of manually specified bounding box coordinates corresponding to the fixed grid layout of the data collection forms. For each scanned form, the script iterated over these coordinates, cropped individual grid cells containing a single character or numeral, and saved each cropped image into its corresponding class-specific directory.

All cropped images were subsequently standardized using a second Python script to ensure uniform input dimensions. Each image was resized to a fixed resolution of 40 × 40 pixels while preserving the original aspect ratio. A scaling factor was computed to fit the handwritten content within the target canvas without distortion. The resized image was then centered, and any remaining space was padded with a white background (RGB value [255, 255, 255]) to produce a uniform square image.

The Python scripts used for automated cropping and resizing, together with the dataset, are freely available in the Mendeley Data repository [1].

## Limitations

The primary limitation of this dataset is that it consists exclusively of isolated letters and numerals, rather than continuous cursive script or complete words. Consequently, while the data is ideal for training classification models, it does not directly support end-to-end Optical Character Recognition (OCR) for full-page documents. Additionally, although the data was collected from diverse demographics, the samples were primarily acquired from the Rangamati and Khagrachhari districts. Handwriting styles specific to Chakma communities across the border in India may be underrepresented.

Furthermore, because the automated cropping pipeline anonymized individual characters by sorting them directly into class-specific directories, metadata linking specific images to individual contributors was not preserved. Consequently, the dataset does not support strict writer-independent data splitting for benchmarking purposes.

## Ethics Statement

This study involved human participants from the indigenous Chakma community. All participants provided their informed consent prior to data collection. The research protocol was carried out in accordance with the Declaration of Helsinki and received approval from the Ethical Review Committee of Green University of Bangladesh (Ref No: GUB/ERC/02/2025).

## CRediT Author Statement

**Conceptualization, Formal Analysis**: All authors

**Data Curation**: Jannatul Ferdeous, Md. Abdul Kayum, and Ahmed Islam

**Methodology**: Jannatul Ferdeous, Md. Abdul Kayum, Ahmed Islam, and Md Roman Nihal

**Software**: Jannatul Ferdeous, Md Abdul Kayum, Ahmed Islam, and Md Roman Nihal

**Validation, Visualization**: Md Roman Nihal

**Supervision**: Muhammad Abul Hasan

**Writing - Original Draft**: Md Roman Nihal and Muhammad Abul Hasan

**Writing - Review & Editing**: ASM Shihabuddin and Muhammad Abul Hasan

**Investigation, Resources**: All authors

**Project Administration**: Muhammad Abul Hasan

## Data Availability

Mendeley DataChakmaLipī Dataset (Original data).
